# Incidence risk and risk factors for postcholecystectomy syndrome: A systematic review and meta-analysis

**DOI:** 10.1097/MD.0000000000047687

**Published:** 2026-02-13

**Authors:** Haining Zhou, Feifei Xuan, Mengxue Liu

**Affiliations:** a Room of Operation, Hangzhou Linping District Integrated Traditional Chinese and Western Medicine Hospital, Hangzhou, Zhejiang, China; b Room of Operation, Shulan (Hangzhou) Hospital, Hangzhou, Zhejiang, China.

**Keywords:** gastrointestinal hormones, laparoscopic cholecystectomy, meta-analysis, postcholecystectomy syndrome, risk factors, treatment outcome

## Abstract

**Background:**

Postcholecystectomy syndrome (PCS) is a clinically significant condition with a variable reported occurrence. This study was conducted to quantitatively synthesize the overall incidence of PCS through meta-analysis and identify its associated risk factors.

**Methods:**

A comprehensive literature search was conducted across multiple databases, including China National Knowledge Infrastructure, Wanfang, Chinese Technical Sciences Periodical Database‌ (VIP), China Biology Medicine, PubMed, Cochrane Library, Embase, and Web of Science, from inception to April 2025. Cohort and case-control studies investigating the incidence and risk factors of PCS following cholecystectomy were included. Statistical analysis was performed using RevMan 5.4 software.

**Results:**

Nine studies involving 2948 cholecystectomy patients were included in the meta-analysis. The pooled analysis demonstrated that the overall incidence of PCS was 3.21% (95% confidence interval: 2.27−4.55%). Preoperative complications were identified as a strong risk factor for PCS (OR = 26.91, 95% confidence interval: 7.87–92.03, *P* < .00001).

**Conclusion:**

PCS presents a considerable clinical risk, particularly in patients with preexisting symptoms. Clinicians should prioritize preoperative assessment, with special attention to symptomatic patients, and implement individualized perioperative management strategies to mitigate PCS risk.

## 1. Introduction

Postcholecystectomy syndrome (PCS) refers to a constellation of symptoms that persist or develop after a cholecystectomy. These symptoms, which include abdominal pain and dyspepsia, have garnered significant clinical attention. With the increasing adoption of laparoscopic cholecystectomy, the incidence of PCS has shown an upward trend, with studies reporting a prevalence rate as high as 15% to 40%.^[1,2]^ PCS not only significantly impairs patients’ quality of life but also imposes a substantial healthcare burden, with associated annual medical expenditures increasing by 2 to 3 times.^[3]^ Of particular concern is that approximately 20% of PCS patients experience symptoms persisting beyond 2 years, with 5% progressing to chronic pain syndrome.^[4]^

A deeper understanding of the risk factors for PCS can enable surgeons to engage in more thorough preoperative discussions with patients, establishing realistic treatment expectations.^[5]^ Identifying key risk factors may help clinicians screen high-risk populations and implement personalized intervention strategies. For instance, patients with preexisting functional gastrointestinal disorders may benefit from enhanced postoperative gastrointestinal function management.^[6]^

Despite the growing recognition of PCS’s clinical importance, there remains a lack of systematic meta-analyses quantifying its incidence and clarifying critical contributing factors through quantitative synthesis. Therefore, this study aims to conduct a rigorous meta-analysis to quantitatively synthesize the overall incidence of PCS in postcholecystectomy patients and to identify its significantly associated risk factors. The findings are intended to provide an evidence-based foundation for clinical practice and guide future research directions.

## 2. Materials and methods

The protocol for this systematic review and meta-analysis was prospectively registered with the International Prospective Register of Systematic Reviews (PROSPERO; registration number: CRD420251121389). The design, implementation, and reporting of this study strictly adhered to the Preferred Reporting Items for Systematic Reviews and Meta-Analyses (PRISMA) guidelines and relevant standards from the Cochrane Handbook for Systematic Reviews of Interventions.

### 2.1. Inclusion and exclusion criteria

Inclusion criteria: Cohort studies or case-control studies, with language restricted to Chinese or English; patients undergoing cholecystectomy; and multivariate logistic regression analysis was used to identify risk factors for PCS in patients after cholecystectomy, with provided odds ratios (ORs) and their 95% confidence intervals (CIs), or data that allowed for conversion and application.

Exclusion criteria: studies with insufficient data, duplicate publications, or unavailable full texts; and reviews, conference abstracts, case reports, or animal experimental studies.

### 2.2. Search strategy

Computerized searches were conducted in the following databases: China National Knowledge Infrastructure, Wanfang, Chinese Technical Sciences Periodical Database‌ (VIP), China Biology Medicine, PubMed, Cochrane Library, Embase, and Web of Science. The search period spanned from inception to April 2025. The search terms included: “Postcholecystectomy Syndrome” OR “PCS,” “risk factor” OR “influencing factor” OR “correlative factor.” A combination of Medical Subject Headings (MeSH) and free-text terms was employed, and the references of included studies were also manually searched.

The PubMed search strategy, as an example, was as follows: ((Postcholecystectomy Syndrome[MeSH Terms]) OR (PCS[Title/Abstract])) AND (((risk factor[Title/Abstract]) OR (influencing factor[Title/Abstract])) OR (correlative factor[Title/Abstract])).

### 2.3. Literature screening, data extraction, and process reliability assessment

The literature screening process initially employed the NoteExpress computerized literature management system for automatic duplicate checking. After deduplication, preliminary screening was conducted by reviewing titles and abstracts. Subsequently, full-text articles were further assessed, and those not meeting the inclusion criteria were excluded. Two researchers independently performed all screening and data extraction steps. To assess the inter-rater reliability and ensure the reproducibility of this process, we calculated Cohen’s kappa (κ) statistic. For the title/abstract screening phase, a random sample of 10% of the records was independently screened by both reviewers, yielding a κ of 0.88. For the full-text screening phase, κ was calculated based on all articles that underwent full-text assessment, resulting in a value of 0.92. Similarly, for key data extraction fields, a random sample of 20% of the included studies was used, yielding a κ of 0.90. All κ values indicated “almost perfect” agreement according to the benchmarks proposed by Landis and Koch. In cases where discrepancies arose between the 2 researchers, attempts were first made to reach a consensus through discussion. If consensus could not be achieved, a third researcher would make the final decision.

The extracted data included authors, publication year, country, study type, sample sizes of the PCS and non-PCS groups, risk factors, and other relevant information.

### 2.4. Quality assessment of literature

Two researchers independently and impartially conducted literature searches, selection, and data extraction based on predetermined inclusion and exclusion criteria. A data extraction template was designed to facilitate the pre-extraction process, thereby ensuring the integrity of the raw data. The 2 researchers independently assessed the quality of the included cohort or case-control studies using the Newcastle–Ottawa Scale.^[7]^ Discrepancies in the quality assessment were resolved through consultation with an internal evidence-based team. The Newcastle–Ottawa Scale framework is designed to evaluate the quality of non-randomized studies in meta-analyses, comprising 2 sections for assessing cohort and case-control studies, respectively. It includes 3 domains: selection of study groups, comparability of groups, and ascertainment of exposure or outcome, ultimately yielding a composite score out of 9. If the 2 researchers disagreed in their evaluations, a third researcher was consulted to reach a final decision.

### 2.5. Statistical methods

The statistical analysis was performed using RevMan 5.4 software. The *I*^2^ statistic was employed to quantify statistical heterogeneity. The choice between a fixed-effect and random-effects model was determined a priori based on the expected degree of clinical and methodological heterogeneity among the included studies. Given the anticipated substantial variations in PCS diagnostic criteria, study populations, surgical techniques, and follow-up durations, a random-effects model (DerSimonian-Laird method) was prespecified as the primary analytical model for all meta-analyses. This model accounts for heterogeneity by assuming that the true effect sizes vary across studies and provides a more conservative and generalizable estimate of the mean effect size and its CI. The fixed-effect model (Mantel–Haenszel method) was considered only in scenarios where studies were deemed sufficiently homogeneous in design, population, and outcome measurement, which was not the case for the primary outcome. The magnitude of heterogeneity between studies was quantitatively assessed using the *I*^2^ statistic along with its 95% CI. *I*^2^ values of 25%, 50%, and 75% were conventionally considered indicative of low, moderate, and high heterogeneity, respectively. A *P*-value < .10 in Cochrane’s *Q* test was regarded as suggesting statistical heterogeneity. These statistics were used to aid in interpreting the variability of pooled results and to justify model selection. All outcome measures included in this study were dichotomous variables; therefore, odds ratios (OR) with 95% CIs were employed as effect measures. The significance level for pooled effects was set at *P* < .05. If 10 or more studies were included, publication bias was evaluated both visually using funnel plots and quantitatively via Egger’s linear regression test. If the number of original studies was limited or the data could not be pooled, a descriptive analysis of the results was conducted.

## 3. Results

### 3.1. Basic characteristics and quality assessment of included studies

**Figure 1.**
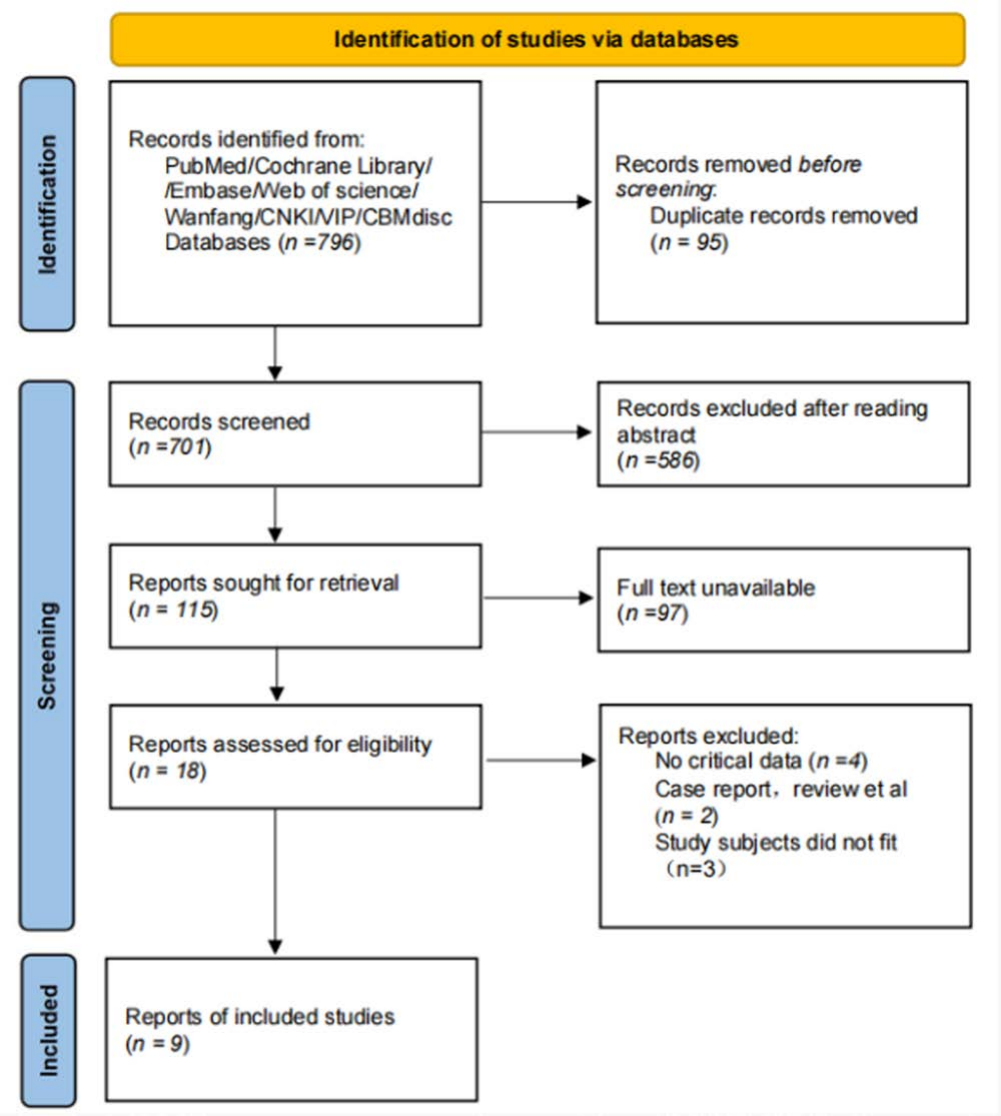
Literature screening flowchart.

A total of 796 articles were retrieved from the database, and 9 studies^[8-16]^ were ultimately included. The literature screening process is shown in Figure 1.

### 3.2. Basic characteristics and methodological quality assessment of included studies

A total of 9 studies^[8-16]^ were included, comprising 6 English-language articles and 3 Chinese-language articles. These studies involved 2948 patients who underwent cholecystectomy, including 651 patients with PCS and 2297 non-PCS patients. The basic characteristics of the included studies are presented in Table 1. Quality assessment of the studies revealed the following scores: 6 studies scored 9 points, 2 studies scored 8 points, and 1 study scored 6 points, as shown in Table 2.

**Table 1:** Basic characteristics of included literature.

Author and year	Sample size		Age (mean ± SD)	PCS incidence	PCS definition	Reported risk factors
	PCS	Non-PCS				
Luo et al (2025)^[8]^	69	262	50.60 ± 13.40	20.85%	Presence of upper abdominal discomfort (bloating, pain) with dyspeptic symptoms (e.g., acid reflux, belching, nausea) 1 mo postoperatively	Preoperative symptoms, age
Li et al (2023)^[9]^	42	118	47.42 ± 8.61	26.25%	New-onset organic or functional biliary disorders, or recurrence of preoperative symptoms after surgery	Preoperative symptoms, gallstones with cholecystitis, traditional open cholecystectomy, elevated serum G-17 levels, elevated serum MOT levels, decreased serum CCK levels
Feng et al (2017)^[10]^	115	201	54.47 ± 13.57	36.39%	Persistence of preoperative symptoms or emergence of new symptoms (e.g., diarrhea, abdominal pain, bloating, constipation, nausea) after cholecystectomy	Preoperative symptoms, anxiety
Shao et al (2025)^[11]^	38	249	59.24 ± 6.86	13.24%	A complex of persistent abdominal symptoms (e.g., pain, fever, jaundice, lower GI discomfort) in patients with a history of cholecystectomy	Common bile duct stones, cholecystitis
Shirah et al (2018)^[12]^	272	1102	37.41 ± 7.12	19.80%	Recurrence of complex symptoms similar to those before surgery (e.g., upper abdominal pain, dyspepsia, with/without jaundice)	–
Treider et al (2023)^[13]^	16	28	8.6 ± 3.9	36.70%	Persistence or occurrence of abdominal symptoms after cholecystectomy	BMI
Shrestha et al (2024)^[14]^	55	72	42.88 ± 13.12	43.30%	Persistence of symptoms after cholecystectomy	Anxiety, moderate to severe dyspepsia
Arora et al (2018)^[15]^	27	180	–	13%	Presence of symptoms (e.g., nausea, vomiting, bloating, jaundice, diarrhea, pain) any time after cholecystectomy	–
Khatana et al (2018)^[16]^	17	85	40.29 ± 10.69	16.66%	A complex of organic and functional disorders after cholecystectomy, resembling preoperative symptoms or new symptoms compatible with biliary disease	–

BMI = body mass index, CCK = cholecystokinin, MOT = motilin, PCS = postcholecystectomy syndrome, SD = standard deviation.

**Table 2:** Quality assessment of included studies.

	Selection			Comparability		Outcome			Total score
	1. Representativeness of the exposed cohort	2. Selection of the nonexposed cohort	3 Ascertainment of exposure	4. Control for important factors	5. Control for additional factors	6. Assessment of outcome	7. Was follow-up long enough for outcomes to occur	8. Adequacy of follow-up	
	PCS patients derived from a well-defined cholecystectomy population	Were non-PCS controls drawn from the same source	Was cholecystectomy clearly confirmed?	Were groups comparable by controlling for age, BMI, or surgical method?	Was there control for other key confounders (e.g., preoperative symptoms)?	Was PCS defined by clear, objective criteria?	Was follow-up ≥3 mo to capture PCS?	Was follow-up rate complete (>80%) or described?	
Luo et al (2025)^[8]^	1	1	1	1	2	1	1	1	9
Li et al (2023)^[9]^	1	1	1	1	2	1	1	1	9
Feng et al (2017)^[10]^	1	1	1	1	2	1	1	1	9
Shao et al (2025)^[11]^	1	1	1	1	2	1	1	1	9
Shirah et al (2018)^[12]^	1	1	0	0	1	1	1	1	6
Treider et al (2023)^[13]^	1	1	0	1	2	1	1	1	8
Shrestha 2024^[14]^	1	1	0	1	2	1	1	1	8
Arora et al (2018)^[15]^	1	1	1	1	2	1	1	1	9
Khatana et al (2018)^[16]^	1	1	1	1	2	1	1	1	9

PCS = postcholecystectomy syndrome.

### 3.3. Meta-analysis results

#### 3.3.1. Incidence of PCS

**Figure 2.**
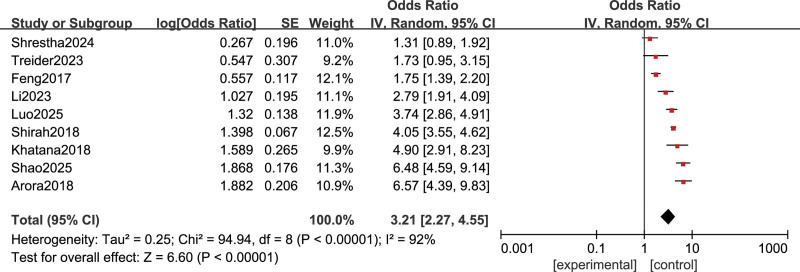
Forest plot of the pooled incidence of PCS. CI = confidence interval, IV = inverse variance, PCS = postcholecystectomy syndrome, SE = standard error.

**Figure 3.**
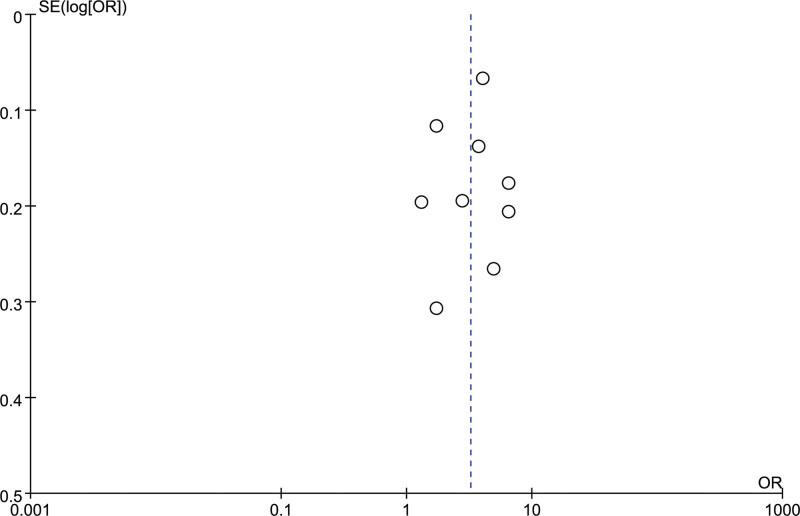
Funnel Plot of the Incidence for PCS. OR = odds ratio, PCS = postcholecystectomy syndrome, SE = standard error.

Nine studies^[8-16]^ reported the incidence of PCS, involving a total of 2948 patients who underwent cholecystectomy. Given potential variations in population characteristics, follow-up duration, or diagnostic criteria for PCS among the included studies, a random-effects model was employed for the pooled analysis. The heterogeneity test confirmed substantial heterogeneity across studies (*I*^2^ = 92%, *P* < .00001). A single-rate meta-analysis was performed to pool the incidence of PCS. The pooled incidence rate of PCS was 3.21% (95% CI: 2.27−4.55%), with significant heterogeneity across studies (*I*^2^ = 99%, *P* < .00001), as shown in Figure 2. In addition, the funnel plot of this analysis is shown in Figure 3.

### 3.4. Preoperative symptoms

**Figure 4.**
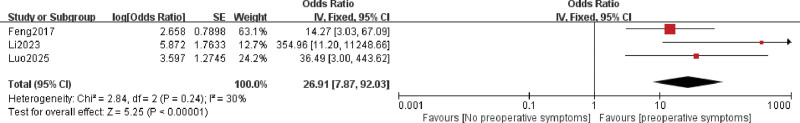
The funnel plot for the analysis of PCS incidence is shown in Figure 3. CI = confidence interval, PCS = postcholecystectomy syndrome, SE = standard error.

Three studies^[8-10]^ reported the impact of preoperative symptoms on PCS. For the factor of preoperative symptoms, we assumed that the studies estimated a common effect size, and thus a fixed-effects model was employed for meta-analysis. Heterogeneity testing indicated low between-study heterogeneity (*I*^2^ = 30%, *P* = .24), supporting the appropriateness of using this model. The results demonstrated that preoperative symptoms are a significant risk factor for PCS (OR = 26.91, 95% CI = 7.87–92.03, *P* < .00001), as shown in Figure 4.

### 3.5. Descriptive analysis results

Due to inconsistencies in the reported risk factors for PCS across studies, with some factors mentioned in only a single or a few publications, it was not feasible to pool the data for meta-analysis. Therefore, this study only provides a descriptive synthesis. The factors were categorized into 4 types:

Demographic factors: Advanced age may be a risk factor, but the strength of evidence is limited. The role of female sex remains unclear.

Clinical factors: Preoperative symptoms and anxiety/depression were significant risk factors, with high effect sizes that remained significant after multivariate adjustment, representing the highest strength of evidence.

Treatment-related factors: Open surgery (as opposed to laparoscopic surgery) and residual cystic duct were identified as important technical risk factors.

Laboratory markers: Some studies reported significant correlations between perioperative gastrointestinal hormone levels (e.g., elevated G-17, decreased cholecystokinin) and the occurrence of PCS, providing potential pathophysiological insights. However, these findings were based on single studies and require independent validation.

In summary, preoperative symptoms and psychological factors currently represent the predictors with the strongest supporting evidence, while other factors, though associated, are supported by weaker evidence and need further confirmation in future studies.

### 3.6. Assessment of publication bias

Begg’s test was performed for the primary outcome (incidence of PCS). The result (*P* = .45) did not indicate significant publication bias. However, it should be noted that the small number of studies included in this meta-analysis may reduce the statistical power of the publication bias test.

## 4. Discussion

This meta-analysis demonstrated a pooled incidence of PCS at 3.21% (95% CI: 2.27–4.55%), indicating that patients face a significant risk of PCS following cholecystectomy. However, there was extremely high heterogeneity between studies (*I*^2^ = 99%), which primarily stems from methodological, demographic, and clinical practice variations. First, methodological heterogeneity is the most significant source. Some studies employed strict Rome IV criteria or objective imaging/laboratory indicators, while others relied on patients’ subjective symptom reports, leading to inconsistent incidence assessments.^[17]^ Additionally, significant differences in study design and follow-up duration contributed to variability. Shorter follow-ups may only capture transient postoperative discomfort, whereas long-term follow-up is necessary to identify the true chronic syndrome. Second, demographic and regional heterogeneity may also play a role. The included studies involved patients from multiple countries such as China, Saudi Arabia, Nepal, and India. Systematic differences in dietary habits, baseline disease profiles, pain sensitivity, and surgical indications may influence the manifestation and incidence of PCS. Third, heterogeneity in clinical practice, such as the ratio of laparoscopic to open surgery, surgical techniques, and perioperative management protocols, could also affect postoperative recovery and PCS occurrence. Due to limited data reporting in the original studies, it was not possible to conduct subgroup analyses or meta-regression for most of the aforementioned factors to quantify their individual contributions. The current risk assessment for PCS remains challenging. On 1 hand, most studies focus on short-term postoperative follow-up, and data on long-term PCS risk are limited, potentially underestimating the true incidence. On the other hand, existing risk prediction models mostly rely on single clinical factors and lack comprehensive assessment tools.^[18,19]^ Future research requires multicenter collaboration to standardize the definition of PCS and extend follow-up duration, while integrating clinical, laboratory, and psychosocial factors to construct more precise risk stratification models for guiding individualized intervention.

Preoperative symptoms were identified as a significant risk factor for PCS, with a high degree of statistical significance (*P* < .00001) and low inter-study heterogeneity (*I*^2^ = 30%), suggesting a relatively robust conclusion. The high OR value indicates that patients with preoperative symptoms have nearly 27 times the risk of developing PCS compared to asymptomatic patients, a finding consistent with the conclusions of Georgescu et al.^[20]^ Their study found that patients with preoperative functional dyspepsia or chronic abdominal pain had a significantly increased incidence of persistent postoperative symptoms. Our results further emphasize the critical role of preoperative symptom assessment in predicting PCS risk. However, the broadly defined risk factor of “preoperative symptoms” requires cautious interpretation. This term encompasses a clinically heterogeneous spectrum in the included studies, ranging from classic biliary colic strongly suggestive of gallbladder disease to nonspecific abdominal discomfort, functional dyspepsia, or symptoms attributable to other underlying conditions.^[21]^ The strong association we observed may reflect that the presence of any preoperative abdominal symptom – regardless of its specific characteristics or etiology – identifies a population at higher risk for persistent postoperative symptoms. Yet, this result cannot distinguish whether the risk stems from misdiagnosis, a preexisting functional gastrointestinal disorder, or specific pathophysiological alterations associated with the original gallbladder dysfunction. Furthermore, the pathophysiology of preoperative symptoms is not fully understood and may involve gallbladder dysfunction, visceral hypersensitivity, or central sensitization, suggesting that the development of PCS is likely multifactorial.^[22]^ Therefore, future research must move beyond this broad classification by employing standardized preoperative symptom assessment tools to capture symptom patterns, location, relationship to meals, and alignment with functional gastrointestinal disorder criteria. This approach is needed to identify specific high-risk symptom profiles for more targeted preoperative counseling and management. Clinicians should also place high importance on evaluating preoperative symptoms. For patients with long-standing or complex symptoms, a more comprehensive preoperative assessment and consideration of individualized surgical decision-making and postoperative management strategies are warranted to reduce PCS risk.

The descriptive analysis in this study reveals the complexity of potential risk factors for PCS. Although we identified several factors possibly associated with PCS, including general patient characteristics (age, BMI), psychological state (anxiety), comorbidities, surgical approach, and laboratory indicators (serum G-17, motilin, and cholecystokinin levels), the evidence for these associations remains preliminary. Inconsistent reporting and the fact that most factors were only explored in 1 or a few studies precluded quantitative meta-analysis, necessitating further validation in future specifically designed studies. Regarding general patient factors, advanced age may increase risk through mechanisms such as decreased gastrointestinal motility and altered visceral sensitivity.^[23]^ Obesity (BMI ≥ 30 kg/m^2^) might affect recovery by promoting chronic low-grade inflammation and altering gastrointestinal hormone secretion patterns.^[24]^ The association with anxiety supports the role of “brain-gut axis” dysregulation in PCS pathogenesis.^[13]^ Among treatment-related factors, traditional open cholecystectomy carries a higher risk compared to laparoscopic surgery, potentially due to greater tissue trauma, a more pronounced inflammatory response, a higher likelihood of adhesions, and a prolonged recovery period.^[25]^ Changes in laboratory indicators provide important clues for understanding the neuroendocrine mechanisms of PCS. Elevated serum gastrin-17 levels may reflect abnormal gastric acid secretion regulation and correlate with postoperative dyspeptic symptoms; increased motilin levels suggest gastrointestinal motility disorders; while decreased cholecystokinin levels might affect the coordinated movement of the biliary and pancreatic systems.^[8]^ These hormonal alterations collectively constitute the “neuroendocrine imbalance” hypothesis for PCS development. However, the interpretation of these potential mechanisms still requires caution.

This study has several important limitations that must be considered when interpreting the findings. The most critical issue is the profound conceptual and clinical heterogeneity among the included studies, which stems primarily from the lack of a standardized definition for PCS. Fundamental differences in diagnostic criteria and wide variations in follow-up duration mean that the included studies measured related but distinct clinical entities. Consequently, the pooled incidence estimate should be interpreted with caution as an aggregate measure of post-cholecystectomy symptom burden across diverse clinical contexts, rather than as a definitive estimate applicable to any specific patient population or clinical setting. Compounding this, the review faces a major threat to validity due to an exceptionally high attrition rate during full-text screening – 97 out of 116 potentially eligible studies were excluded because full texts were unavailable. This compromises the systematic comprehensiveness of the work, introduces a high risk of selection bias, and suggests the final included studies may not be representative of all relevant evidence, thereby fundamentally constraining the robustness and generalizability of our conclusions. The meta-analysis of risk factors is also methodologically limited; the analysis for preoperative symptoms was based on only 3 studies with inconsistent definitions of both exposure and outcome, resulting in an imprecise effect estimate that reflects considerable uncertainty even under a random-effects model. Similarly, the small number of studies and inconsistent reporting for other potential risk factors precluded their quantitative synthesis, which limits the strength of the conclusions. Furthermore, due to heterogeneous reporting in the primary studies, we could not separately analyze “structural” and “functional” PCS subtypes – a significant constraint on the clinical applicability of our findings, as management strategies differ fundamentally for these etiologies. Additional limitations include a search restricted to Chinese and English databases and publications, which may introduce language bias and lead to the omission of relevant studies, and the observational design of the included studies, which limits causal inference due to potential residual confounding. Finally, planned subgroup analyses were not feasible due to insufficient data reporting in the primary studies. Collectively, these limitations underscore the need for future research to adopt standardized diagnostic criteria for PCS, improve data accessibility, and conduct well-designed prospective studies or individual patient data meta-analyses.

In summary, this meta-analysis confirms a high incidence risk of PCS, which is particularly significant for patients with preoperative clinical symptoms. Additionally, potential risk factors such as age, BMI, surgical approach, and gastrointestinal hormone levels show some association with PCS, but require more research for validation. These findings suggest that clinicians should emphasize preoperative assessment, pay special attention to patients with existing clinical symptoms, and consider adopting individualized perioperative management strategies. Future research should involve more rigorously designed, adequately powered prospective studies to establish standardized PCS diagnostic criteria and risk assessment systems. This will help further clarify the predictive value and mechanisms of various risk factors, providing more reliable evidence for the prevention and precise intervention of PCS.

## Author contributions

**Conceptualization:** Haining Zhou, Feifei Xuan.

**Data curation:** Haining Zhou, Feifei Xuan, Mengxue Liu.

**Formal analysis:** Mengxue Liu.

**Writing – original draft:** Haining Zhou, Feifei Xuan, Mengxue Liu.

**Writing – review & editing:** Haining Zhou, Feifei Xuan, Mengxue Liu.
